# Capillary malformation–arteriovenous malformation syndrome (CM-AVM): a systematic review of cerebrovascular manifestations

**DOI:** 10.1007/s00381-025-07089-5

**Published:** 2025-12-16

**Authors:** Matteo Palermo, Alessandro Olivi, Carmelo Lucio Sturiale

**Affiliations:** Department of Neurosurgery, Fondazione Policlinico Universitario A. Gemelli IRCCS, Università Cattolica del Sacro Cuore, L.go A. Gemelli 8, 00168 Rome, Italy

**Keywords:** Capillary malformation, High-flow vascular malformations, RASA1, EPHB4

## Abstract

**Background:**

Capillary malformation–arteriovenous malformation (CM-AVM) syndrome is a rare genetic disorder characterized by cutaneous capillary malformations and fast-flow vascular lesions, including arteriovenous malformations (AVMs) and arteriovenous fistulas (AVFs). CM-AVM is caused by mutations in RASA1 and EPHB4, leading to aberrant Ras-MAPK signaling.

**Methods:**

A systematic search of PubMed and Scopus was conducted for studies published until June 2025. The inclusion criteria were studies reporting cerebrovascular malformations in genetically confirmed CM-AVM cases. A total of 37 studies were included in the final analysis.

**Results:**

The review included 148 patients diagnosed with CM-AVM, with 86% carrying RASA1 mutations and 14% carrying EPHB4 mutations. The most common cerebrovascular lesions were pial AVFs (43.3%) and AVMs (36.0%), with a notable distinction between the two genetic subtypes. RASA1 mutations were associated with a broader range of lesions, including AVMs, pAVFs, and vGaMs, whereas EPHB4 mutations were predominantly linked to vGaMs. Nearly 25% of patients required endovascular embolization, and 5.3% underwent surgery. A significant difference in the cerebrovascular phenotype was observed between RASA1 and EPHB4 mutations, with the latter group presenting a narrower vascular phenotype.

**Conclusion:**

This review highlights the crucial need for screening cerebrovascular anomalies in CM-AVM patients due to potential misdiagnosis with HHT. Genetic testing is essential for confirmation, but regular imaging and clinical evaluation are key to detecting vascular lesions early, preventing severe neurological complications. Further research into additional genetic mutations may improve diagnostic accuracy and management strategies.

## Introduction

Capillary malformation–arteriovenous malformation (CM-AVM) syndrome is a rare autosomal dominant vascular disorder characterized by multiple small cutaneous capillary malformations (CMs) associated with fast-flow vascular lesions such as arteriovenous malformations (AVMs) or arteriovenous fistulas (AVFs) [1–3]. Patients typically present with numerous pink-to-red capillary lesions on the skin that blanch with pressure and often have a surrounding pale halo. These multifocal CMs are most commonly distributed on the face, limbs, and trunk and may be present at birth or arise during childhood. In approximately 15–30% of reported cases, one or more fast-flow vascular anomalies are observed in the skin, muscle, bone, or visceral organs; intracranial AVMs or AVFs have also been reported in a subset of patients [4, 5].

Genetic studies have established that CM-AVM is caused by pathogenic variants in at least two genes. The initial form, now termed CM-AVM type 1 (CM-AVM1), was first described in 2003 and is due to heterozygous loss-of-function mutations in RASA1, which encodes p120-RasGAP. RASA1 is a negative regulator of the RAS/MAPK pathway, and its mutation leads to aberrant Ras-MAPK signaling in vascular endothelial cells. More recently, a second genetic subtype called CM-AVM type 2 (CM-AVM2) was identified, caused by mutations in EPHB4. EPHB4 is a tyrosine kinase receptor in the ephrin B2/EphB4 signaling axis that also converges on Ras-MAPK and angiogenesis pathways. Together, RASA1 and EPHB4 mutations account for the majority of CM-AVM cases. Both are inherited in an autosomal dominant pattern with high but incomplete penetrance (approximately 90–95%); de novo mutations and mosaic cases are well documented [6–9].

Clinically, CM-AVM can be subtle and is sometimes misdiagnosed as other vascular disorders. In particular, hereditary hemorrhagic telangiectasia (HHT) is an important differential diagnosis. HHT is another autosomal dominant vascular condition due to mutations in the genes *ENG* or *ACVRL1* that also features mucocutaneous telangiectasias, recurrent nosebleeds, and visceral AVMs [4, 10–16]. However, CM-AVM is pathogenetically distinct from HHT, and patients usually have the capillary stain lesions and lack some of the classic HHT organ manifestations (e.g., pulmonary AVMs). Genetic testing is therefore critical when the phenotype is suggestive; identifying a *RASA1* or *EPHB4* mutation confirms the diagnosis of CM-AVM and rules out HHT. In this paper, we focus exclusively on genetically confirmed CM-AVM cases (CM-AVM1 and CM-AVM2) in order to define the syndrome’s specific cerebrovascular phenotype [4, 5, 17]. We present a systematic review and of reported CM-AVM cases, highlighting the frequency and nature of central nervous system (CNS) vascular lesions in this population. The goal of our review is to clarify the range of intracranial vascular anomalies that occur in CM-AVM, analyze potential genotype–phenotype correlations between RASA1 vs. EPHB4 variants, and discuss implications for diagnosis and management of these patients [18].

## Methods

**Fig. 1:**
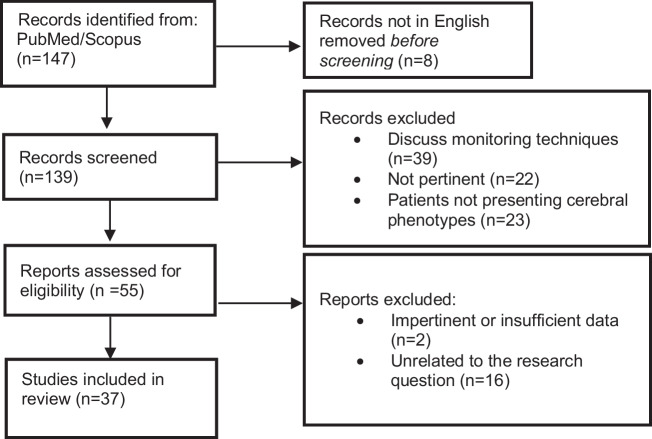
PRISMA flow diagram—study selection process for the systematic review

This review was performed according to the PRISMA (Preferred reporting items for systematic reviews and meta-analyses) 2020 guidelines [19]. The PICO framework (Population: CM-AVM patients; Intervention: HHT patients; Comparison: cerebrovascular malformations; Outcome: prevalence) was used to formulate the research question (Fig. 1).

### Search strategy

Two authors (CLS and MP) performed a comprehensive search on PubMed/MEDLINE and Scopus databases to identify relevant studies discussing cerebrovascular malformations different from AVMs in HHT patients using the search terms: “(*RASA1 or EPHB4*) AND (*capillary malformation OR CM OR CM-AVM*)”. The search was updated to June 20th 2025, with no time limit. A forward search on references of the retrieved articles was also performed to increase the search power.

### Study selection

The search was restricted to peer-reviewed, English-language studies that included quantitative data. We included studies that reported on cerebrovascular malformations in patients with a genetically confirmed diagnosis of CM-AVM (either type 1 or type 2). For the purposes of this study, we only included patients with a definitive diagnosis of CM-AVM presenting cerebrovascular anomalies. We excluded individuals with diagnoses of other vascular malformation syndromes, including Parkes Weber Syndrome, Klippel-Trénaunay-Weber Syndrome (KTWS), Hereditary Neurocutaneous Angioma, and Lymphatic Malformation 7 (LM7), among others, to ensure that our analysis was focused specifically on CM-AVM and its associated genetic mutations. Animal and pre-clinical studies were excluded, as were review articles and studies lacking explicit data. Two authors (CLS and MP) independently screened the titles and abstracts of all articles identified through the search algorithm and selected studies based on the predefined inclusion and exclusion criteria.

After excluding ineligible articles, the full texts of the remaining studies were reviewed to confirm eligibility using the same criteria (Fig. 1). Any disagreements were resolved during a consensus meeting through joint reassessment of the article and the extracted data.

### Data extraction

For each eligible study, we extracted author and year of publication, number of patients, mutated gene (RASA1 or EPHB4), and specific genetic mutation (cDNA and/or protein notation). Additionally, we extracted the age at diagnosis, sex, ethnicity, cerebrovascular phenotype, and type of treatment. For the cerebrovascular phenotype, we documented the presence or absence of the following lesions: arteriovenous malformation (AVM), arteriovenous fistula (AVF), vein of Galen malformation (VGaM), brain aneurysm, and cerebral cavernous malformation (Table 1). We also attempted to distinguish between pial and dural AVFs whenever sufficient detail was provided. AVMs were included regardless of whether they were cerebral or spinal in location.

**Table 1:** Study and patient characteristics—overview of included studies, genetic mutations, and clinical data

Author, year	No. pts	Genetic mutation		Mutation	Syndrome	Age of diagnosis (years)	Sex	Ethnicity	AVM	pAVF	VGaM	Aneurysm	Cavernoma	Treatment	Outcome
		RASA1	EPHB4												
Romano, 2025	5	1		c.1986_1989delAAAG (p.Lys664Alafs*13)	CM-AVM1	6	F	Caucasian	0	1	0	0	0	Embolization	N/A
		1		c.656C > G (p.Ser219*)	CM-AVM1	11	M	Caucasian	0	0	1	0	0	Embolization	N/A
			1	c.874A > G (p.Ser292Gly)	CM-AVM2	11	M	Caucasian	0	0	1	0	0	Embolization	N/A
			1	c.3G > A (p.Met1?)	CM-AVM2	17	M	Caucasian	0	0	1	0	0	Embolization	N/A
			1	c.2231G > A (p.Arg744His)	CM-AVM2	3	F	Caucasian	1	1	0	0	0	Conservative	N/A
Tyson, 2025	1		1	c.175G > A, p.Glu59Lys	CM-AVM2	1	M	N/A	0	1	0	0	0	Embolization	Good clinical stats; no neurological deficit; stable after closure
Chen, 2024	2	1		N/A	CM-AVM1	N/A	N/A	Asian	1	N/A	0	0	0	N/A	N/A
			1	N/A	CM-AVM2	N/A	N/A	Asian	1	N/A			0	N/A	N/A
Weinberger, 2023	1	1		N/A	CM-AVM1	6	M	N/A	0	0	0	1	0	Surgery	Excellent neurological recovery; complete occlusion; no recurrence at early follow-up
LoPresti, 2023	2	1		N/A	CM-AVM1	N/A	N/A	N/A	1	0	0	0	0	Surgery	N/A
		1		N/A	CM-AVM1	N/A	N/A	N/A	1	0	0	0	0	Surgery	N/A
Guimaraes, 2022	1	1		N/A	CM-AVM1	0.1	M			0	0	1	0	Embolization	N/A
Wang, 2022	2	1		c.1280G > A (missense variant)	CM-AVM1	28	M		1	0	0	0	0	N/A	N/A
		1		c.3007G > A (missense variant)	CM-AVM1	27	F		1	0	0	0	0	N/A	N/A
Amyere, 2017	3		1	c.1990G > A (p.E664K)	CM-AVM2	N/A	F	Caucasian	0	0	1	0	0	N/A	N/A
			1	c.2484 + 1G > A	CM-AVM2	N/A	M	Caucasian	0	0	1	0	0	N/A	N/A
			1	N/A	CM-AVM2	N/A	F	Caucasian	0	1	0	0	0	N/A	N/A
Wooderchak-Donahue, 2019	2		1	c.1423-6G > A (p.Gly475Thrfs*39)	CM-AVM2	17	M	N/A	0	0	1	0	0	N/A	N/A
			1	c.443G > A (p.Arg148Gln)	CM-AVM2	13	N/A	N/A	1	0	0	0	0	N/A	N/A
Haefliger, 2021	3	1		N/A	CM-AVM1	N/A	N/A	N/A	1	0	0	0	0	N/A	Resolution of sensorimotor symptoms after embolisation
		1		N/A	CM-AVM1	N/A	N/A	N/A	0	0	1	0	0	N/A	N/A
		1		N/A	CM-AVM1	N/A	N/A	N/A	0	1	0	0	0	Embolization	N/A
Mizutani, 2021	1	1		N/A	CM-AVM1	11	F	N/A	1	0	0	0	0	N/A	N/A
Kumai, 2021	1	1		c.1571_1572insTT(p.Cys525TyrfsTer20)	CM-AVM1 + COL4A2 syndrome	N/A	N/A	N/A	0	1	0	0	0	Embolization	N/A
Hajjam, 2019	1	1		c.556_562del (p.Leu186Glufs*)	CM-AVM1	28	M	N/A	1	0	0	0	0	Embolization	N/A
Valdivielso-Ramos, 2020	5	1		N/A	CM-AVM1	N/A	N/A	N/A	1	0	0	0	0	N/A	N/A
		1		N/A	CM-AVM1	N/A	N/A	N/A	1	0	0	0	0	N/A	N/A
		1		N/A	CM-AVM1	N/A	N/A	N/A	1	0	0	0	0	N/A	N/A
		1		N/A	CM-AVM1	N/A	N/A	N/A	1	0	0	0	0	N/A	N/A
		1		N/A	CM-AVM1	N/A	N/A	N/A	0	1	0	0	0	N/A	N/A
Grillner, 2016	3	1		c.2125C > T (exon 16)	CM-AVM1	0.5	F	N/A	0	0	1	0	0	Embolization	N/A
		1		c.2707C > T (exon 21)	CM-AVM1	Preterm	M	N/A	0	1	0	0	0	Embolization	N/A
		1		c.1279C > T (exon 9)	CM-AVM1	Preterm	F	N/A	0	1	0	0	0	Embolization	N/A
Saliou, 2017	14	1		c.2513dup, p.Asn838Lysfs*2	CM-AVM1	Preterm	M	N/A	0	1	0	0	0	Embolization	N/A
		1		c.1455del, p.Gly487Glufs*11	CM-AVM1	Preterm	M	N/A	0	1	0	0	0	Embolization	N/A
		1		c.556_562del, p.Leu186Glufs*3	CM-AVM1	0.5	M	N/A	0	1	0	0	0	Embolization	N/A
		1		c.2707C > T, p.Arg903*	CM-AVM1	Preterm	M	N/A	0	1	0	0	0	Embolization	N/A
		1		c.1509del, p.Gln503Hisfs*17	CM-AVM1	Preterm	F	N/A	0	1	0	0	0	Embolization	N/A
		1		c.1102 + 2 T > C, p.? (splice site mutation)	CM-AVM1	0.8	M	N/A	0	1	0		0	Embolization	N/A
		1		c.261_262del, p.Gly89Argfs*22	CM-AVM1	20	F	N/A	0	1	0	0	0	Embolization	N/A
		1		c.261del, p.Gly89Glufs*7	CM-AVM1	Preterm	M	N/A	0	1	0	0	0	Embolization	N/A
		1		c.2422C > T, p.Gln808*	CM-AVM1	Preterm	F	N/A	0	1	0	0	0	Embolization	N/A
		1		c.2703del, p.Leu902Phefs*9	CM-AVM1	Preterm	M	N/A	0	1	0	0	0	Embolization	N/A
		1		c.1567_1568del, p.Ser523Cysfs*9	CM-AVM1	Preterm	F	N/A	0	1	0	0	0	Embolization	N/A
		1		c.1594dup, p.Asp532Glyfs*2	CM-AVM1	Preterm	M	N/A	0	1	0	0	0	Embolization	N/A
		1		c.383_384del, p.Leu128Argfs*29	CM-AVM1	Preterm	F	N/A	0	1	0	0	0	Embolization	N/A
		1		c.2441del, p.Leu814X + c.296C > T, p.Ala99Val	CM-AVM1	Preterm	M	N/A	0	1	0	0	0	Embolization	N/A
Overcash, 2015	2	1		(c.1698 + 2dupT)	CM-AVM1	38	F	N/A	1	1	0	0	0	Embolization; Surgery	Uncomplicated postpartum course; alive and stable
		1		(c.1698 + 2dupT)	CM-AVM1	Preterm	M	N/A	1	0	0	0	0	Surgery	Survived and discharged
Ryu, 2020	1	1		N/A	CM-AVM1	Preterm	M	N/A	1	1	0	0	0	Embolization	Complete obliteration; no treatment-related complications; clinical course did not worsen during 10-month follow-up
Moreno-Estebanez, 2020	1	1		1579_1582delGTCTp (Val527fs)	CM-AVM1	25	M	N/A	1	0	0	0	0	N/A	Complete resolution of neurologic symptoms
Plumptre, 2019	1	1		N/A	CM-AVM1	1.5	M	Caucasian	0	1	0	0	0	Embolization	Permanent diplegia
Revencu, 2020	1	1		c.1879A > T, p.(Lys627*)	CM-AVM1	N/A	N/A	N/A	1	1	0	0	0	N/A	N/A
Moteki, 2019	2	1		c.2925 + 1G > T	CM-AVM1	0.8	N/A	N/A	0	1	0	0	0	Embolization	Good clinical and radiologic outcomes reported – improvement of cardiac/CSF-flow issues; probands survive
		1		c.724delGinsCT, p.Gly242LeufsTer23	CM-AVM1	0.3	N/A	N/A	0	1	0	0	0	Embolization	N/A
Wooderchak-Donahue, 2018	11	1		c.475_476del, p.(Leu159Glyfs*20)	CM-AVM1	5	F	N/A	1	0	0	0	0	N/A	N/A
		1		c.1493_1494del, p.(Glu498Glyfs*2)	CM-AVM1	5	M	N/A	1	0	0	0	0	N/A	N/A
		1		c.1513_1514insAA, p.(Ile505Lysfs*16)	CM-AVM1	27	F	N/A	1	0	0	0	0	N/A	N/A
		1		c.1771dup, p.(Arg591Profs*11)	CM-AVM1	5	M	N/A	1	0	0	0	0	N/A	N/A
		1		c.2131C > T, p.(Arg711*)	CM-AVM1	1	M	N/A	1	0	0	0	0	N/A	N/A
		1		c.2225C > A, p.(Ser742*)	CM-AVM1	49	F	N/A	1	0	0	0	0	N/A	N/A
		1		c.2365C > T, p.(Arg789*)	CM-AVM1	13	M	N/A	1	0	0	0	0	N/A	N/A
		1		c.2603 + 1G > A	CM-AVM1	13	F	N/A	1	0	0	0	0	N/A	N/A
		1		c.2603 + 1G > A	CM-AVM1	Preterm	F	N/A	1	0	0	0	0	N/A	N/A
		1		c.2603 + 4del	CM-AVM1	2	M	N/A	1	0	0	0	0	N/A	N/A
		1		c.2604G > T	CM-AVM1	2	F	N/A	1	0	0	0	0	N/A	N/A
Vivanti, 2018	2		1	c.570dupG (p.His191Alafs*32)	CM-AVM2	0.2	N/A	N/A	0	0	1	0	0	Embolization	N/A
			1	c.2484 + 1G > T (p.Met814_Val829del)	CM-AVM2	0.5	N/A	N/A	0	0	1	0	0	Embolization	N/A
Lapinski, 2018	1	1		c.1703G > A, p.Trp568*	CM-AVM1	10	F	Hispanic	0	1	0	0	0	N/A	N/A
Chugh, 2017	2	1		N/A	CM-AVM1	3	M	African-American	0	1	0	0	0	Surgery	At 14-month follow-up: neurologically stable
		1		N/A	CM-AVM1	0.8	F	African-American	0	1	0	0	0	Surgery	At 4-year follow-up only subtle residual proptosis, neurologically intact
Ashlee, 2013	1	1		c.2084A > T; p.His695Le	CM-AVM1	3	F	N/A	0	3	0	0	1	Embolization	No long-term neurological outcome
Revencu, 2013+ Thiex, 2010	19	1		c.475 476del, p.Leu159Glyfs ∗ 2	CM-AVM1	N/A	N/A	N/A	0	1	0	0	0	N/A	N/A
		1		c.492C > G p.Tyr164 ∗	CM-AVM1	N/A	N/A	N/A	1	0	0	0	0	N/A	N/A
		1		c.1192C > T p.Arg398*	CM-AVM1	N/A	N/A	N/A	1	0	0	0	0	N/A	N/A
		1		c.1386 1387insCT p.Ile463Leufs ∗ 2	CM-AVM1	6	F	N/A	1	0	0	0	0	N/A	N/A
		1		c.1453 + 1del Splicing	CM-AVM1	6	M	N/A	0	1	0	0	0	N/A	N/A
		1		c.1589 T > A p.Val530Asp	CM-AVM1	N/A	N/A	N/A	1	0	0	0	0	N/A	N/A
		1		c.1666 1698 + 15del Splicing	CM-AVM1	36	F	N/A	1	1	0	0	0	N/A	N/A
		1		c.1717C > T p.Gln573*	CM-AVM1	34	M	N/A	1	0	0	0	0	N/A	N/A
		1		c.2125C > T p.Arg709 ∗	CM-AVM1	N/A	N/A	N/A	0	0	1	0	0	N/A	N/A
		1		c.2131C > T p.Arg711 ∗	CM-AVM1	N/A	N/A	N/A	0	1	0	0	0	N/A	N/A
		1		c.2131C > T p.Arg711 ∗	CM-AVM1	N/A	N/A	N/A	1	0	0	0	0	N/A	N/A
		1		c.2329G > T p.Glu777 ∗	CM-AVM1	6	M	N/A	0	1	0	0	0	N/A	N/A
		1		c.2603 + 2dup Splicing	CM-AVM1	N/A	N/A	N/A	1	0	0	0	0	N/A	N/A
		1		c.2707C > T p.Arg903 ∗	CM-AVM1	N/A	N/A	N/A	1	0	0	0	0	N/A	N/A
		1		c.2925 + 5G > C Splicing	CM-AVM1	N/A	N/A	N/A	1	0	0	0	0	N/A	N/A
		1		c.2977del p.Arg993Valfs ∗ 3	CM-AVM1	N/A	N/A	N/A	0	0	1	0	0	N/A	N/A
		1		c.3024del p.Glu1008Aspfs ∗ 16	CM-AVM1	N/A	N/A	N/A	0	0	1	0	0	N/A	N/A
		1		c.[3028C > T; 64G > T] p.[Arg1010 ∗; Gly22Cys]	CM-AVM1	N/A	N/A	N/A	1	0	0	0	0	N/A	N/A
Català, 2013	1	1		N/A	CM-AVM1	1.5	F	N/A	0	1	0	0	0	Embolization	N/A
Eerola, 2003	1	1		c.475_476delCT	CM-AVM1	N/A	N/A	N/A	1	0	0	0	0	N/A	N/A
Wooderchak-Donahue, 2011	3	1		c.1491_1492delAG	CM-AVM1	N/A	N/A	N/A	1	0	0	0	0	N/A	N/A
		1		c.1771_2insC	CM-AVM1	N/A	N/A	N/A	1	0	0	0	0	N/A	N/A
		1		c.2225C > A	CM-AVM1	N/A	N/A	N/A	1	0	0	0	0	N/A	N/A
Walcott, 2012	2	1		N/A	CM-AVM1	5	N/A	N/A	0	1	0	0	0	Embolization	N/A
		1		N/A	CM-AVM1	4	N/A	N/A	0	1	0	0	0	Embolization + Surgery	N/A
Revencu, 2008	10	1		c.1192C > T (p.Arg398X)	CM-AVM1	N/A	N/A	N/A	1	0	0	0	0	N/A	N/A
		1		c.1208dupC (p.Thr404AsnfsX14)	CM-AVM1	N/A	N/A	N/A	1	0	0	0	0	N/A	N/A
		1		c.1490 T > G (p.Leu497X)	CM-AVM1	N/A	N/A	N/A	1	0	0	0	0	N/A	N/A
		1		c.1636C > T (p.Gln546X)	CM-AVM1	N/A	N/A	N/A	1	0	0	0	0	N/A	N/A
		1		c.1682_1683dup (Pro562LeufsX9)	CM-AVM1	N/A	N/A	N/A	1	0	0	0	0	N/A	N/A
		1		c.2125C > T (p.Arg709X)	CM-AVM1	N/A	N/A	N/A	1	0	0	0	0	N/A	N/A
		1		c.2288A > T (p.Glu763Val)	CM-AVM1	N/A	N/A	N/A	0	0	0	0	1	N/A	N/A
		1		c.2341G > T (p.Glu781X)	CM-AVM1	N/A	N/A	N/A	1	0	0	0	0	N/A	N/A
		1		C > T (p.Arg789X)	CM-AVM1	N/A	N/A	N/A	1	0	0	0	0	N/A	N/A
		1		c.2532_2536delTTTAA (p.Leu845ThrfsX38)	CM-AVM1	N/A	N/A	N/A	0	0	0	0	1	N/A	N/A
Tas, 2022	38	1		N/A	CM-AVM1	N/A	N/A	N/A	0	1	0	0	0	N/A	N/A
		1		N/A	CM-AVM1	N/A	N/A	N/A	0	1	0	0	0	N/A	N/A
		1		N/A	CM-AVM1	N/A	N/A	N/A	0	1	0	0	0	N/A	N/A
		1		N/A	CM-AVM1	N/A	N/A	N/A	0	1	0	0	0	N/A	N/A
		1		N/A	CM-AVM1	N/A	N/A	N/A	0	1	0	0	0	N/A	N/A
		1		N/A	CM-AVM1	N/A	N/A	N/A	0	1	0	0	0	N/A	N/A
		1		N/A	CM-AVM1	N/A	N/A	N/A	0	1	0	0	0	N/A	N/A
		1		N/A	CM-AVM1	N/A	N/A	N/A	0	1	0	0	0	N/A	N/A
		1		N/A	CM-AVM1	N/A	N/A	N/A	0	1	0	0	0	N/A	N/A
		1		N/A	CM-AVM1	N/A	N/A	N/A	0	1	0	0	0	N/A	N/A
		1		N/A	CM-AVM1	N/A	N/A	N/A	0	1	0	0	0	N/A	N/A
		1		N/A	CM-AVM1	N/A	N/A	N/A	0	1	0	0	0	N/A	N/A
		1		N/A	CM-AVM1	N/A	N/A	N/A	0	1	0	0	0	N/A	N/A
		1		N/A	CM-AVM1	N/A	N/A	N/A	0	1	0	0	0	N/A	N/A
		1		N/A	CM-AVM1	N/A	N/A	N/A	0	1	0	0	0	N/A	N/A
		1		N/A	CM-AVM1	N/A	N/A	N/A	0	1	0	0	0	N/A	N/A
		1		N/A	CM-AVM1	N/A	N/A	N/A	0	1	0	0	0	N/A	N/A
		1		N/A	CM-AVM1	N/A	N/A	N/A	0	1	0	0	0	N/A	N/A
		1		N/A	CM-AVM1	N/A	N/A	N/A	0	1	0	0	0	N/A	N/A
		1		N/A	CM-AVM1	N/A	N/A	N/A	0	1	0	0	0	N/A	N/A
		1		N/A	CM-AVM1	N/A	N/A	N/A	0	1	0	0	0	N/A	N/A
		1		N/A	CM-AVM1	N/A	N/A	N/A	0	1	0	0	0	N/A	N/A
		1		N/A	CM-AVM1	N/A	N/A	N/A	0	1	0	0	0	N/A	N/A
		1		N/A	CM-AVM1	N/A	N/A	N/A	0	1	0	0	0	N/A	N/A
		1		N/A	CM-AVM1	N/A	N/A	N/A	0	1	0	0	0	N/A	N/A
		1		N/A	CM-AVM1	N/A	N/A	N/A	0	1	0	0	0	N/A	N/A
		1		N/A	CM-AVM1	N/A	N/A	N/A	0	0	1	0	0	N/A	N/A
		1		N/A	CM-AVM1	N/A	N/A	N/A	0	0	1	0	0	N/A	N/A
		1		N/A	CM-AVM1	N/A	N/A	N/A	0	1		0	0	N/A	N/A
			1	N/A	CM-AVM2	N/A	N/A	N/A	0	0	1	0	0	N/A	N/A
			1	N/A	CM-AVM2	N/A	N/A	N/A	0	0	1	0	0	N/A	N/A
			1	N/A	CM-AVM2	N/A	N/A	N/A	0	0	1	0	0	N/A	N/A
			1	N/A	CM-AVM2	N/A	N/A	N/A	0	0	1	0	0	N/A	N/A
			1	N/A	CM-AVM2	N/A	N/A	N/A	0	0	1	0	0	N/A	N/A
			1	N/A	CM-AVM2	N/A	N/A	N/A	0	0	1	0	0	N/A	N/A
			1	N/A	CM-AVM2	N/A	N/A	N/A	0	0	1	0	0	N/A	N/A
			1	N/A	CM-AVM2	N/A	N/A	N/A	0	0	1	0	0	N/A	N/A
			1	N/A	CM-AVM2	N/A	N/A	N/A	0	0	1	0	0	N/A	N/A
Kwong, 2015	2	1		c.2119C > T; p.Arg707Cys	CM-AVM1	Preterm	N/A	N/A	0	0	1	0	0	N/A	Neonate died on day 5 due to intractable high-output cardiac failure despite embolization
		1		N/A	CM-AVM1	Preterm	N/A	N/A	0	0	1	0	0	N/A	Pterminated after multidisciplinary counseling
Paramasivam, 2013	1	1		N/A	CM-AVM1	5	M	N/A	0	1	0	0	0	N/A	N/A
TOTALS	148	127	21	-	CM-AVM1 (112); CM-AVM2 (21)	-	M (34); F (26)	Hispanic (1); Caucasian (9); Asian (2); African-American (2)	54	63	26	2	3	Embolization (36); Surgery (7); Conservative (1); Embolization + Surgery (1)	-

### Risk of bias

**Fig. 2:**
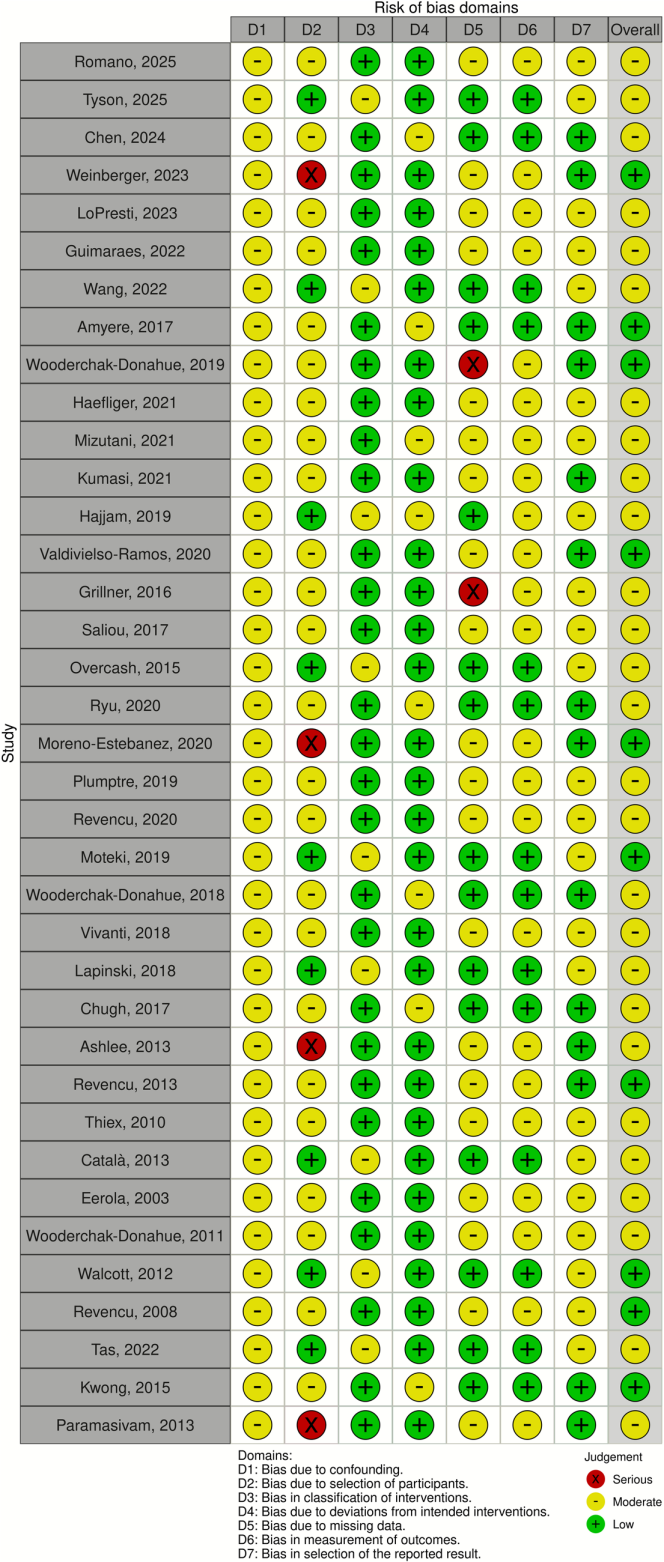
Risk of bias summary—ROBINS-I assessment of included studies

The ROBINS-I V2 (Risk of bias in non-randomized studies – of interventions, version 2) assessment tool along with the Robins application (https://mcguinlu.shinyapps.io/robvis/) was used to evaluate study quality through visual representations (Fig. 2).

## Results

The initial search retrieved 147 records from PubMed and Scopus. Before screening, eight non-English records were excluded. During the screening phase, 84 records were excluded for the following reasons: discussion of monitoring techniques without mentioning cerebrovascular phenotypes (*n* = 39), lack of relevance to the topic (*n* = 22), or absence of cerebral phenotypes in the patient population (*n* = 23). Of the 55 reports assessed for eligibility, 17 were excluded due to impertinent (e.g., epistaxis management, hepatic involvement, pregnancy outcomes, or genetic counseling) or insufficient quantitative data (*n* = 2), as they were unrelated to the research question (*n* = 16). Ultimately, 37 studies were included in the final analysis (Fig. 1; Table 1). The quality of these studies was assessed using the ROBINS-I version 2 tool to evaluate the risk of bias (Fig. 2). The study selection process was documented according to the PRISMA 2020 guidelines, with a flow diagram illustrating the phases of identification, screening, eligibility assessment, and final inclusion (Fig. 1).

### Qualitative analysis (systematic review)

A total of 37 studies published between 2003 and 2025 were included, encompassing 148 patients diagnosed with capillary malformation–arteriovenous malformation syndrome (CM-AVM) and genetically confirmed to carry either RASA1 or EPHB4 mutations (Table 1) [1–11, 14, 17, 18, 20–43]. Most patients had undergone presymptomatic genetic testing or presented with a neurovascular phenotype prompting imaging. For the type of mutation, when available, refer to Table 1 for detailed reporting [1–11, 14, 17, 18, 20–43].

Genetic analysis revealed 127 patients (86.0%) carried a RASA1 mutation, while 21 (14.0%) had a mutation in EPHB4. Accordingly, 127 patients were classified as CM-AVM1 and 21 as CM-AVM2 based on genotype [1–11, 14, 17, 18, 20–43]. Sex was reported in 60 individuals: 34 males (56.5%) and 26 females (43.5%) [6, 8, 11, 21, 22]. Ethnicity was reported in 14 patients, including Caucasian (*n* = 9), Asian (*n* = 2), African-American (*n* = 2), and Hispanic (*n* = 1).

Among the cerebrovascular malformations reported, the most frequently observed lesions were pAVFs, present in 63 patients (43.3%). Notably, all AVFs were pial, and no dural AVFs were identified. This was followed by AVMs in 54 patients (36.0%), and vGaMs in 26 patients (17.3%). Less common findings included intracranial aneurysms (*n* = 2; 1.3%) and cavernomas (*n* = 3; 2.0%).

As regards treatment, a total of 36 patients (24.7%) underwent endovascular embolization, while 7 (5.3%) underwent surgery, 1 (0.7%) was treated conservatively, and 1 (0.7%) received a combined approach [1–11, 14, 17, 18, 20–43].

When stratified by gene mutation, notable differences emerged in the distribution of cerebrovascular malformations. The most common cerebrovascular malformation in patients with RASA1 mutation was pAVF, reported in 61 patients (48.03%), followed by AVMs in 54 patients (42.52%), and vGaMs in 12 patients (9.44%). Rare findings included cavernomas (*n* = 2, 1.57%) and intracranial aneurysms (*n* = 1; 0.79%) (Table 2) [1–11, 14, 17, 18, 20–43].

**Table 2:** Cerebrovascular lesions in CM-AVM1 (RASA1)—frequency of brain lesions in RASA1-mutated patients

Type of cerebrovascular malformation	Number of patients	Percentage (%)
pAVF	61	48.03%
AVM	54	42.52%
VGaM	12	9.44%
Aneurysm	1	0.79%
Cavernoma	2	1.57%

In contrast, the EPHB4 subgroup demonstrated a narrower cerebrovascular phenotype dominated by vein of Galen malformations, which were present in 14 patients (66.7%). Only two patients (9.5%) exhibited pial AVFs, while no brain AVMs were observed in this group. Intracranial aneurysms and cavernomas were each reported in one patient (4.8%), respectively (Table 3) [1–11, 14, 17, 18, 20–43].

**Table 3:** Cerebrovascular lesions in CM-AVM2 (EPHB4)—frequency of brain lesions in EPHB4-mutated patients

Type of cerebrovascular malformation	Number of patients	Percentage (%)
VGaM	14	66.7%
pAVF	2	9.5%
AVM	0	0.0%
Aneurysm	1	4.8%
Cavernoma	1	4.8%

## Discussion

### Cerebrovascular differences with HHT

**Fig. 3:**
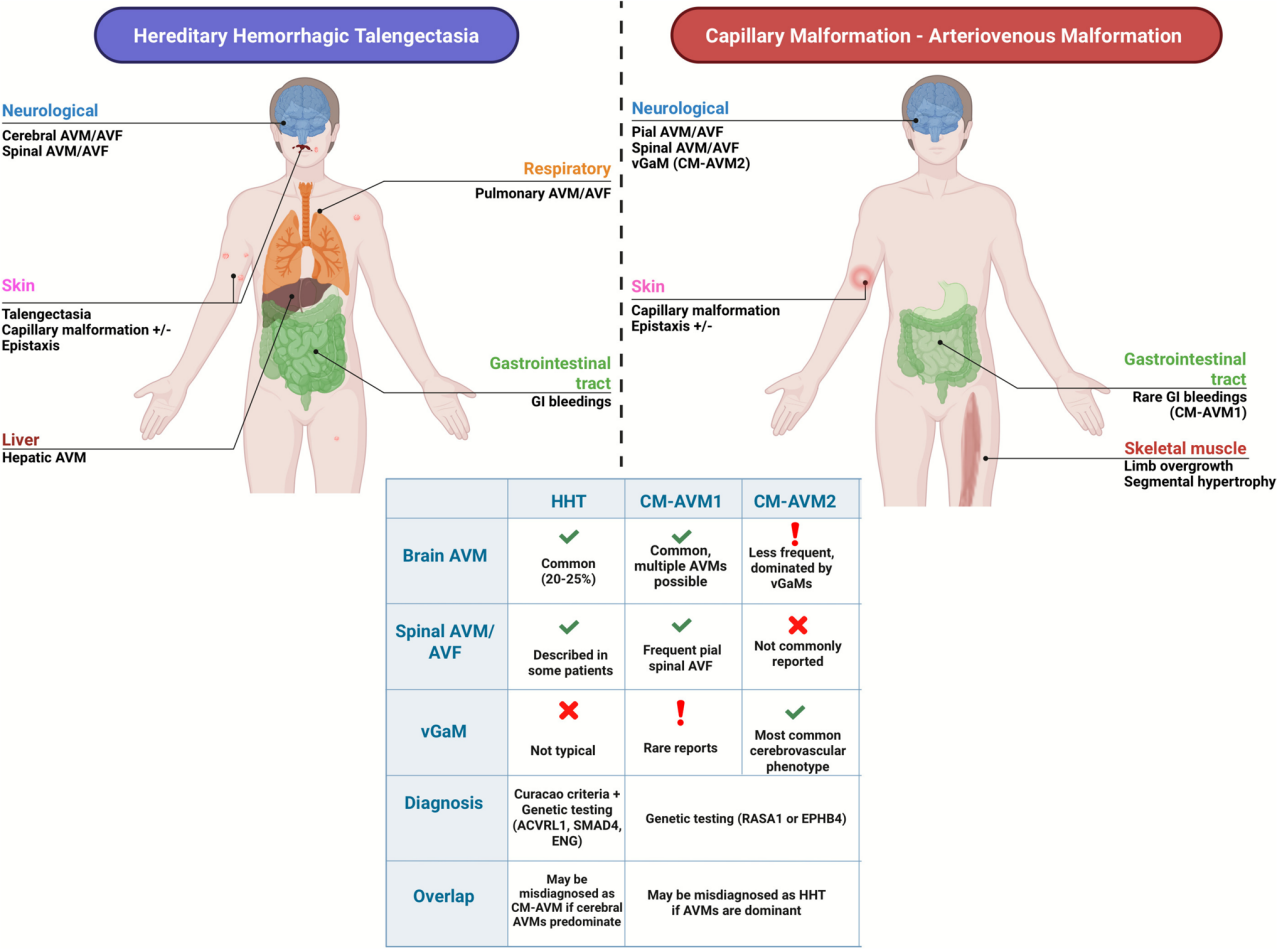
Graphical representation of common lesions and summary table of differential findings

Our systematic review highlights distinct cerebrovascular phenotypes associated with RASA1 versus EPHB4 mutations in CM-AVM syndrome (Fig. 3). RASA1-related CM-AVM typically exhibited a broad spectrum of high-flow lesions, with nearly half of these patients harboring pAVFs and a similar proportion presenting with AVMs. A smaller subset exhibited vGaMs, and rare occurrences of intracranial aneurysms or cavernomas were also noted [1–11, 14, 17, 18, 20–43]. In contrast, EPHB4-related CM-AVM demonstrated a narrower cerebrovascular phenotype, predominantly characterized by vGaMs [2, 14, 17, 26].

These differences have significant clinical implications. The prominence of neonatal vGaM in EPHB4-mutated cases means CM-AVM2 often presents early, even prenatally or in the neonatal period, with high-output cardiac failure or hydrocephalus, necessitating urgent intervention. In this review, nearly 25% of CM-AVM underwent endovascular embolization, indicating that these malformations were retained at elevated hemorrhagic risk [1, 7, 9, 15, 18, 25, 28, 30, 32, 34, 37, 44]. While CM-AVM1 patients often present with multiple AVMs or AVFs, which may require interventions over time, the CM-AVM2 subgroup, though critically ill in infancy due to vGaM, did not develop additional brain AVMs later in life. This suggests that, once the primary congenital shunt (vGaM) is addressed, EPHB4 patients may have fewer intracranial sequelae than RASA1 patients, although further confirming longitudinal data are needed [37, 39, 42, 43].

This study identifies differences in cerebrovascular phenotypes between CM-AVM1 and CM-AVM2, although the study does not have sufficient power to demonstrate significant differences. However, CM-AVM2 patients appear to be more prone to HHT-like features, such as epistaxis and mucocutaneous telangiectasia, which can initially lead to misclassification as HHT [45], 46. Despite these overlapping features, RASA1 mutations may still be more frequently associated with intracranial AVMs than EPHB4 mutations, although this remains statistically unproven [18].

Regarding management, a multidisciplinary approach is essential for CM-AVM patients, involving dermatology, genetics, neurology, neurosurgery, and interventional radiology teams. While treatment for cutaneous lesions is generally conservative or involves laser therapy for cosmetic purposes, the primary focus remains on the management of high-flow lesions, including AVMs, AVFs, and vGaMs [4, 14, 20, 24, 26, 29, 32]. For pediatric cases, early intervention is critical, especially for neonates presenting with vGaM, to prevent life-threatening complications such as heart failure and brain injury. Genetic counseling is also an essential aspect of care, particularly due to the autosomal dominant inheritance of CM-AVM, which warrants family screening and evaluation [9].

### Extra-cerebrovascular findings different from HHT

CM-AVM1 and CM-AVM2 present several distinguishing characteristics compared with HHT. While both CM-AVM and HHT share overlapping features, most notably a predisposition to cutaneous vascular lesions and cerebral AVMs, which can lead to intracranial hemorrhage, they also differ in multiple important aspects. Indeed, epistaxis and mucocutaneous telangiectasia, hallmark features of HHT, have also been reported in a subset of CM-AVM patients, especially those with EPHB4 mutations [1, 3, 5, 9, 11, 36–38] (Fig. 3). These overlaps have led to patients with RASA1 or EPHB4 mutations occasionally being misdiagnosed as HHT when they present with recurrent nosebleeds or red skin spots. Given the clinical convergence, genetic testing panels for unexplained vascular anomalies now often include RASA1 and EPHB4 alongside classic HHT genes (ENG, ACVRL1, SMAD4) [1, 3, 5, 9, 11, 36–38].

Despite the overlap, significant differences exist between CM-AVM and HHT in their manifestations and pattern of organ involvement: HHT is characterized by telangiectases on mucosal surfaces and acral skin, commonly on the lips, oral cavity, face, and fingertips. These lesions are usually small (pinpoint to a few millimeters), appear in adolescence or adulthood, and are often countable [22, 26, 34]. In contrast, CM-AVM1/2 patients typically have capillary malformations (CMs) that present as pink-red macules or port-wine stain-like birthmarks on the skin. These CMs are often present in early childhood (sometimes at birth) and tend to be on the trunk and extremities (arms, legs, torso) rather than the fingertips [6, 10, 39]. Multiple CMs are common; some individuals have too numerous to count red macules across broad skin areas, which is more suggestive of CM-AVM than HHT. Moreover, some CM-AVM lesions can be larger (1–2 cm or more), port-wine stains, or associated with underlying fast-flow shunts, whereas HHT telangiectases are usually tiny and superficial [4, 17, 24, 42].

Recurrent spontaneous nosebleeds are the classic symptom of HHT, present in over 90% of adults with HHT. In CM-AVM, epistaxis is not a consistent feature; most RASA1 or EPHB4 mutation carriers do not experience significant nosebleeds. However, there are rare exceptions where CM-AVM patients develop HHT-like epistaxis due to nasal telangiectases [7, 27, 28, 30, 33]. For instance, El Hajjam et al. reported a RASA1-mutated patient with typical nasal telangiectasia and severe epistaxis, initially suggestive of HHT. Such cases are unusual, and the absence of chronic epistaxis in a patient with multiple AVMs favors CM-AVM over HHT in the differential diagnosis [20, 30, 38].

Perhaps the most pronounced difference is the involvement of internal organs. HHT almost invariably involves visceral arteriovenous shunts: pulmonary AVMs occur in roughly 15–50% of HHT patients, and hepatic AVMs or telangiectasias are also very common, often leading to high-output cardiac failure or biliary disease in later life. In stark contrast, traditional CM-AVM1/2 has not been associated with pulmonary or hepatic AVMs in the vast majority of cases [1, 3, 5, 9, 11, 36–38]. A study of over 300 individuals with RASA1 mutations found no lung or liver AVMs, highlighting that these organs are typically spared in CM-AVM. However, not all 300 were diagnosed with CM-AVM syndrome; most had RASA1 mutation polymorphisms, with only a subset confirmed to have the syndrome. This means routine screening for lung or liver AVMs, a cornerstone in HHT management, has not been considered necessary in RASA1/EPHB4 syndromes. Only recently have a few notable exceptions blurred this distinction: two RASA1-positive patients were reported with large pulmonary and hepatic AVMs mimicking HHT, and separate case series have shown that EPHB4 mutations can occasionally lead to HHT-like hepatic vascular abnormalities in older adults [39, 41]. These rare cases suggest some overlap in extreme phenotypes; however, the routine presence of multiple lung and liver AVMs remains a hallmark of HHT and not CM-AVM.

Both disorders can present with cerebral and spinal AVMs, but their patterns differ. Cerebral AVMs affect only 10% of patients with HHT (with higher prevalence in ENG-mutation HHT1 than in HHT2), and HHT-related brain AVMs are usually sporadic in number. In contrast, among individuals with RASA1 mutations in our review, *all of whom were selected based on the presence of cerebrovascular phenotypes*, over 80% had at least one intracranial fast-flow lesion (AVM, pAVF, or vGaM), often manifesting in infancy or childhood. Furthermore, vGaMs, a severe neonatal form of AVM, are frequently seen in EPHB4-related CM-AVM2, while being never reported in HHT cases [1, 3, 5, 9, 11, 36–38].

In summary, HHT patients require screening for pulmonary/hepatic AVMs and management of hemorrhagic telangiectases, whereas CM-AVM patients (RASA1 or EPHB4) should be closely monitored for treatable cerebral or spinal AVMs and high-flow shunts, but typically do not need routine lung or liver intervention. However, genetic testing plays a pivotal role in resolving diagnostic ambiguity, helping to uncover the underlying cause and ensuring that patients receive appropriate counseling and surveillance.

### Genetic spectrum of CM-AVM

The spectrum of RASA1 and EPHB4 mutations identified in the 150 CM-AVM patients is diverse, but a unifying theme is that most are predicted to cause loss of function of the respective protein. The mutations compiled from 37 studies (Table 1) include numerous frameshift insertions/deletions, nonsense variants, and splice-site mutations, as well as a smaller number of missense changes [1, 3, 5, 9, 11, 36–38]. In our review, no obvious “hotspots” emerged in either gene; mutations were distributed throughout the coding sequences of RASA1 (which spans about 25 exons) and EPHB4 (about 19 exons). Recurrent mutations were few, suggesting most families have private pathogenic variants, although some recurrent changes (e.g., RASA1 c.475\_476del, c.2707C > T (p.Arg903\*)), have been noted in multiple unrelated patients, hinting at possible mutation-prone sites in the genome.

This variability is in line with the hypothesis that stochastic events or “second hits” contribute to lesion development in CM-AVM [18, 31, 42]. A two-hit model has been proposed wherein an inherited germline RASA1 mutation is followed by a somatic “second hit” in local endothelial cells, leading to a complete loss of RASA1 function in that clone and the formation of a focal AVM. Supporting this, some AVM tissue samples in CM-AVM1 have demonstrated somatic mutations in the second RASA1 allele [18, 31, 42]. This model helps explain why only a subset of mutation carriers develop large AVMs: in our series, although all patients were genetically confirmed, it is known from broader studies that many individuals with RASA1 mutations may have only cutaneous CMs and never manifest a symptomatic AVM. Future research on lesional tissue DNA could further elucidate how commonly a second-hit mechanism operates in both RASA1 and EPHB4-related malformations [18, 31, 42].

### CM-AVM pathophysiology

From a pathophysiologic standpoint, RASA1 and EPHB4 mutations converge on aberrant RAS/ERK signaling in vascular development. RASA1 encodes p120-RasGAP, a negative regulator of Ras; haploinsufficiency of RASA1 leads to excessive Ras-MAPK pathway activity in endothelial cells, which is thought to disrupt normal capillary plexus maturation and angiogenic remodeling. EPHB4 is a tyrosine kinase receptor expressed on venous endothelial cells; EPHB4 plays a crucial role in distinguishing venous vs. arterial vasculature during embryogenesis [6, 17, 21, 42, 43]. Loss-of-function variants in EPHB4 likely impair downstream signaling that intersects with the Ras-MAPK and VEGF pathways, resulting in failure of proper vessel differentiation and stability. Indeed, EPHB4-related CM-AVM2 has been shown to “mimic” RASA1-related disease at the molecular level by deregulating RAS-MAPK signaling in a similar fashion [3, 5, 7, 27, 36]. This shared pathway disruption explains why RASA1 and EPHB4 mutations produce overlapping clinical syndromes, despite one being a cytoplasmic RasGAP and the other a cell-surface receptor. By contrast, the genes implicated in HHT (ENG, ACVRL1, SMAD4, and GDF2) encode components of the TGF-β/BMP9 signaling axis that governs vascular quiescence and integrity [3, 5, 7, 27, 36]. The differing molecular pathways likely underlie some of the clinical differences discussed above.

### Limitations

There are important limitations to recognize in our review and in the current literature. All data on CM-AVM come from case reports and small series, introducing publication bias. This means the true incidence of brain AVMs/AVFs in unselected CM-AVM populations might be lower, or potentially some mild cases go unreported. Of note, our review specifically included patients with documented cerebrovascular involvement and therefore does not represent the full spectrum of affected individuals. Furthermore, not all published cases had comprehensive workups; some older reports might not have performed brain MRI on every patient, so milder asymptomatic lesions could be under-detected. A related limitation is the overlap with HHT in clinical features; it is possible that a few patients initially diagnosed as “atypical HHT” in the past were actually CM-AVM2 before EPHB4 was discovered. As genetic testing becomes more routine, misdiagnosis will decrease, and clearer genotype-stratified data should emerge. Additionally, the discovery of *EPHB4* mutations opened the question of other genes in the same pathway: indeed, mutations in *EFNB2* (the ligand for EphB4) have recently been implicated in vGaMs in humans. It is conceivable that future studies will identify other genetic contributors that cause overlapping phenotypes of capillary malformations with AVMs. Finally, quantitative meta-analytic techniques were not performed due to insufficiently homogeneous data.

## Conclusion

In conclusion, our review highlights distinct cerebrovascular phenotypes in RASA1 and EPHB4 mutations in CM-AVM. Screening for cerebrovascular anomalies and genetic testing are crucial for differentiating CM-AVM from HHT. Early identification and a multidisciplinary approach are key to effective management.

## Data Availability

Available upon reasonable request.

## Abbreviations

CM-AVM: Capillary malformation–arteriovenous malformation
AVM: Arteriovenous malformation
AVF: Arteriovenous fistula
pAVF: Pial arteriovenous fistula
vGaM: Vein of Galen malformation
HHT: Hereditary hemorrhagic telangiectasia
PRISMA: Preferred reporting items for systematic reviews and meta-analyses
ROBINS-I: Risk of bias in non-randomized studies of interventions
MAPK: Mitogen-activated protein kinase
